# What samples are suitable for monitoring antimicrobial-resistant genes? Using NGS technology, a comparison between eDNA and mrDNA analysis from environmental water

**DOI:** 10.3389/fmicb.2023.954783

**Published:** 2023-12-14

**Authors:** Kahoko Takeda-Nishikawa, Rajaguru Palanichamy, Naoki Miyazato, Takayoshi Suzuki

**Affiliations:** ^1^Faculty of Commerce, Chuo University, Tokyo, Japan; ^2^Department of Biotechnology, Central University of Tamil Nadu, Thiruvarur, India; ^3^National Institute of Technology (KOSEN), Gunma College, Maebashi, Japan; ^4^Division of Molecular Target and Gene Therapy Products, National Institute of Health Sciences, Kawasaki, Japan

**Keywords:** antimicrobial resistance (AMR), beta-lactam, antimicrobial-resistant genes, environmental DNA (eDNA), AMR pollution

## Abstract

**Introduction:**

The rise in antimicrobial resistance (AMR) that is affecting humans, animals, and the environment, compromises the human immune system and represents a significant threat to public health. Regarding the impact on water sanitation, the risk that antimicrobial-resistant genes (ARGs) and antimicrobial-resistant bacteria in surface water in cities pose to human health remains unclear. To determine the prevalence of AMR in environmental surface water in Japan, we used DNA sequencing techniques on environmental water DNA (eDNA) and the DNA of multidrug-resistant bacteria (mrDNA).

**Methods:**

The eDNA was extracted from four surface water samples obtained from the Tokyo area and subjected to high- throughput next-generation DNA sequencing using Illumina-derived shotgun metagenome analysis. The sequence data were analyzed using the AmrPlusPlus pipeline and the MEGARes database. Multidrug-resistant bacteria were isolated using a culture-based method from water samples and were screened by antimicrobial susceptibility testing (for tetracycline, ampicillin-sulbactam, amikacin, levofloxacin, imipenem, and clarithromycin). Of the 284 isolates, 22 were identified as multidrug-resistant bacteria. The mrDNA was sequenced using the Oxford nanopore MinION system and analyzed by NanoARG, a web service for detecting and contextualizing ARGs.

**Results and discussion:**

The results from eDNA and mrDNA revealed that ARGs encoding beta-lactams and multidrug resistance, including multidrug efflux pump genes, were frequently detected in surface water samples. However, mrDNA also revealed many sequence reads from multidrug-resistant bacteria, as well as nonspecific ARGs, whereas eDNA revealed specific ARGs such as pathogenic OXA-type and New Delhi metallo (NDM)-beta-lactamase ARGs.

**Conclusion:**

To estimate potential AMR pollution, our findings suggested that eDNA is preferable for detecting pathogen ARGs.

## Introduction

The world is facing a growing threat of AMR. In fact, according to an OECD report (OECD, 2018), AMR rates have been increasing in OECD countries from 2005 to 2015. Approximately 35% of infections are already resistant in Turkey, Korea, and Greece. It is estimated that about 2.4 million people could die in Europe, North America, and Australia without prompt and effective action, due to AMR between 2015 and 2050. To address concerns about the impact of AMR on human health, the World Health Organization introduced the “One Health” concept in 2015 (Mackenzie and Jeggo, 2019). This approach involves designing and implementing programs encompassing clinical control, food safety, and environmental health. In response to the One Health approach, the government of Japan released the “National Action Plan on Antimicrobial Resistance (AMR).” This plan emphasized the “One Health surveillance system,” which involves researching to monitor AMR and residual antimicrobials in aquatic environments.

It is important to be mindful of the health of our aquatic environment, as studies have shown the presence of antimicrobial-resistant Gram-negative bacteria in and around drinking water sources (Young and Jesudason, 1990; Liguori et al., 2022). Moreover, increasing levels of ARGs have been reported in environmental water (Zhou et al., 2017). River in megacity such as the Hudson River mouth (Young et al., 2013) in NY and Tama River (Iwane et al., 2001) in Tokyo were detected AMR. The runoff from the hotspot of AMR, such as a hospital, also impacts environmental AMR pollution. The pathogen bacteria in hospitals were detected the same in the river (Hassoun-Kheir et al., 2020). In recent years, AMR pollution means detecting both AMR and ARGs (Nakayama et al., 2017; Shin et al., 2023), and AMR pollution in rivers is a growing public health challenge with ARGs regarded as a critical emerging contaminant (Liguori et al., 2022; Taing et al., 2022). In these situations, we need to manage AMR pollution in environmental water. To manage AMR pollution, it is inevitable to monitor AMR contamination in environmental water regularly, and the monitoring method should be accurate, inexpensive, and easily detected for AMR contamination. To address this need, we compared two different samples to detect environmental AMR. One was eDNA extracted DNA from the environmental water, and another was mrDNA extracted DNA from multidrug-resistant bacteria.

Previous studies have commonly used culture-based methods for isolating target bacteria on general or selective media, followed by assessing for ARGs. It is a reliable method to detect ARGs from mrDNA because most multidrug-resistant bacteria have several series of ARGs. However, culture-based analysis using mrDNA consumes more time than eDNA analysis. The eDNA analysis does not need the process of the culture of bacteria. In addition, as mentioned above, eDNA is including ARGs from uncultured microbiota in environmental water. On the other hand, mrDNA gave us the ARGs information from only culturable bacteria.

Initially, eDNA was used to estimate the presence of fish species in river ecological surveys (Takahara et al., 2012). As part of the meta-barcoding approach, it is used for continuous biodiversity monitoring in managing ecosystem-based fisheries (Miya et al., 2020). Furthermore, eDNA has been applied to metagenomics for various environmental creatures, such as microbes, plankton, fungi, and invertebrates (Ruppert et al., 2019). The Sterivex-filter is commonly used in these types of surveys (Wong et al., 2020). Thus, eDNA is developing for broader use in academic research. In addition, NGSs have been supported by databases of ARGs recently. The NGS platform of nanopore sequencer, the MinION system, was released in 2014. It provides long sequence reads averaging 5 kb in length (Mikheyev and Tin, 2014). The analytical tool for ARGs, NanoARG, which follows the DeepARG-LS model, a novel deep learning approach, has been fitted for the use of nanopore-derived metagenomics data involving long reads (Arango-Argoty et al., 2018). According to Arango-Argoty and colleagues (Arango-Argoty et al., 2019), NanoARG estimates the abundance of ARG classes and groups them by their copy numbers. To enable a comparison of the abundance of ARGs across samples, the copy number of ARGs is normalized to total gigabase pairs of the sample to obtain the relative ARG abundance (Ma et al., 2016). The combination of the MinION sequencer and NanoARG is a powerful metagenomic tool for environmental surveys of AMR.

Another NGS platform, the MiniSeq Sequencing System by Illumina, Inc. The MiniSeq system is an array-based technology capable of reading approximately 150–300 bp length. The Illumina sequencing platform was applied to the human gut microbiome study (Qin et al., 2010). The AMR sequencing data by The MiniSeq system can submit databases of ARGs, such as the AmrPlusPlus pipeline, using the MEGARes database (Lakin et al., 2017). MEGARes is a hand-curated database and annotation structure for ARGs that offers a foundation for developing high-throughput classifiers and conducting hierarchical statistical analyses of big data. As MEGARes can be browsed as a stand-alone resource on its affiliated website, it is helpful for users to estimate AMR pollution using bioinformatics. In this study, we chose NGS tools suitable for two different DNA types to investigate the detection of AMR contamination in rivers. In practice, eDNA from environmental water was subjected to Miniseq sequencing, and the dataset was analyzed with the AmrPlusPlus pipeline using MEGARes. By contrast, the mrDNA extracted from isolated multidrug-resistant bacteria was subjected to MinION sequencing, and the dataset was analyzed with the NanoARG.

This study mainly compares two types of DNA, eDNA and mrDNA. Then, we discussed suitable samples for estimating ARGs in an urban river. Samples were collected from aquatic environments close to residents, such as rivers and ponds in the Tokyo area. Our findings provide initial insight into the most appropriate and user-friendly samples for estimating AMR pollution accurately.

## Materials and methods

### Environmental water DNA (eDNA) samples

**Figure 1:**
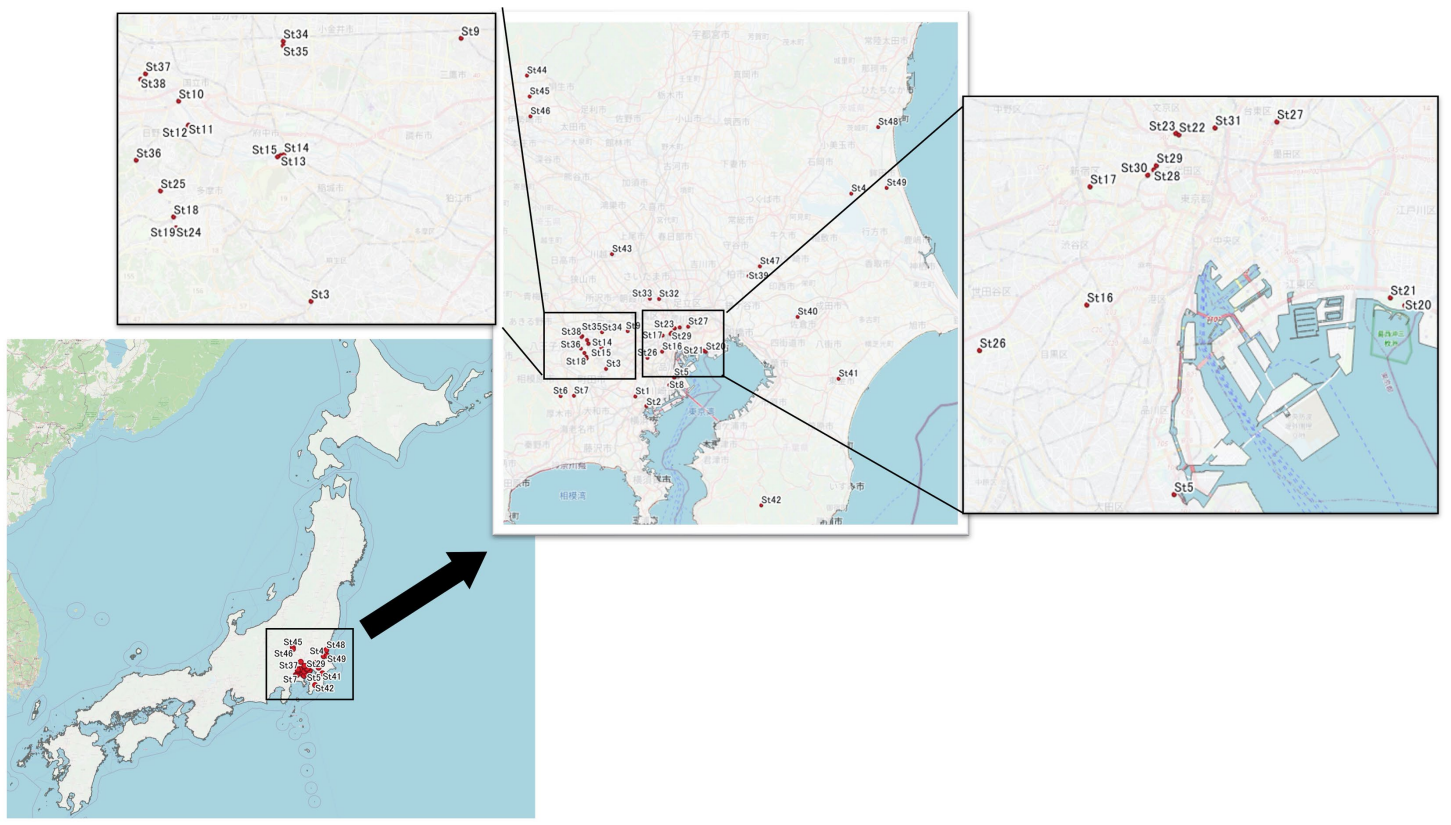
Sampling sites. We collected water samples from 49 sites, Tokyo and near prefecture (Saitama, Gunma, Kanagawa, Chiba, and Ibaraki) were included. However, mainly we focused on Tokyo area. Water samples were collected from river or pond. Asterisk mark (*) showed eDNA sampling points; St. 9, 10, 12, and 31.

For eDNA, we collected water 15 cm below the surface of a river or pond in public park, and placed it in a sterile 1-L disposal flask that was stored in a cooler bag containing an ice pack prior to filtration. Sampling locations are indicated by the four points at St 9, 10, 12, and 31 (Figure 1). Sequential filtering of the water samples was performed within 3 h of collection. First, the water samples were filtered through a pre-filter (Y100A047A, Advantec®, diameter: 47 mm, pore size: 10 μm) to remove all contamination (including large particles, such as algae and debris) except for the target bacteria. Then, 1 L of sample waters were filtered through a Sterivex-GP filter (Millipore; pore size: 0.22 μm), which trapped the bacterial cells and exo-cellular DNA for subsequent eDNA extraction. Cells stored at ˗80°C prior to analysis.

### Extraction of eDNA and shotgun metagenome analysis

Firstly, eDNA (St.9, 10, 12, and 31) was eluted from Sterivex-GP filter using lysis buffer in the MoBio PowerWater DNA Isolation Kit (MoBio Laboratories, Germany), following the manufacturer’s protocol. Most of our water samples contained low levels of bacterial DNA, making it difficult to extract the required quantity of eDNA using 1-L of water. The eDNA isolated from four water samples was therefore pooled.

Shotgun metagenome analysis was conducted on the eDNA. First, paired-end sequencing libraries (2 × 150 bp) were prepared using the Nextra XT DNA Library Preparation Kit and Nextra XT Index Kit (Illumina, Inc.). Next, the eDNA library was pooled, and multiplexing libraries were constructed. Then, these libraries were applied to the MiniSeq High Output Reagent Kit for the MiniSeq system (Illumina, Inc.) for sequencing. After obtaining the sequence data, the dataset in FASTA format was submitted to AmrPlusPlus. AmrPusPlus is a Galaxy-based metagenomics pipeline that identifies and characterizes resistance genes within sequence data using the MEGARes database (https://megares.meglab.org/; Doster et al., 2020) that contains the sequence data of approximately 4,000 hand-curated ARGs. This program identifies all ARGs within the sequence data using a user-specified gene fraction threshold. The gene fraction is defined as the proportion of nucleotides in a reference sequence to which at least one read from the sequence data is aligned. The AmrPlusPlus pipeline can filter out genes with gene fractions below a user-defined threshold to prevent false-positive ARG identifications from short-read sequence data. We used the default settings. Counts of aligned reads were recorded at the gene, group, mechanism, and class levels and were provided as output. The class level indicated the major molecular category of resistance to different classes of antimicrobial drugs (i.e., tetracyclines, beta-lactams, glycopeptides). The next level down, the mechanism level, corresponds to the molecular mechanism that confers resistance to antibiotics. For example, fluoroquinolone-resistant DNA topoisomerases represent a resistance mechanism within the fluoroquinolones class. The next level down, the group level, is categorized based on information contained within the gene or operon. The group annotation provides information on the major gene category, for example, the beta-lactamase gene contains group annotations corresponding to the gene names to which they are associated, e.g., SHV or TEM beta-lactamase. The resistome indicates the counts of resistance genes in our data, and a class-level profile was produced according to the counts in the gene-level profile using AmrPlusPlus.

### Isolation of microbes from water samples

Water samples were gathered from 49 sites (Figure 1) on non-rainy days and bacteria were isolated using cultivation-based methods. Similar to eDNA isolation, first, water samples were filtered through a pre-filter (Y100A047A, Advantec^®^, diameter: 47 mm, pore size: 10 mm) and 0.1 mL of each filtrate was inoculated onto R2A agar (Becton Dickinson) and BBL™ Standard Methods Agar (Tryptone Glucose Yeast Agar) (Becton Dickinson) plates for bacterial isolation. The plates were incubated at 25°C for 2–4 days. Each colony grown on the plate was transferred aseptically into nutrient broth (Becton Dickinson) medium. The pure liquid culture was incubated at 37°C overnight and used for antimicrobial susceptibility testing.

### Antimicrobial susceptibility testing

For screening, antimicrobial susceptibility testing was performed using the BBL™ Sensi-Disc™ susceptibility testing kit (Becton Dickinson) on Mueller–Hinton agar (Becton Dickinson), according to the manufacturer’s instructions. The antimicrobial agents amikacin (30 μg), tetracycline (30 μg), ampicillin-sulbactam (10/10 μg), levofloxacin (5 μg), imipenem (10 μg), and clarithromycin (15 μg) were used. All isolates were obtained from a single colony, and overnight cultures were used for testing. In brief, after inoculating with pure overnight cultures, the plates were incubated at 37°C overnight and then inhibition zone diameters were measured to classify the degree of susceptibility. All isolates were classified as sensitive (S), intermediate (I), or resistant (R) according to the manufacturer’s standard for *Enterobacteriaceae*, *Pseudomonas aeruginosa*, *Acinetobacter*, and *Staphylococci*. These standards were based on the Clinical and Laboratory Standards Institute [CLSI] (Weinstein, 2018) breakpoints.

### DNA extraction and MinION sequencing

Based on the antimicrobial-resistance profiles of the isolated microbes, 22 strains were selected as High-resistant bacteria, exhibiting non-susceptibility to more than two antimicrobials. The mrDNA was extracted and purified from pure bacterial suspensions using the Gentra Puregene Yeast/Bact kit (Qiagen) and quantified using the Qubit fluorometer (Invitrogen). According to the manufacturer’s instructions, a library was prepared using a Rapid PCR Barcoding kit (SQK-RPB004, Oxford Nanopore Technologies; ONT). The sample was analyzed on the MinION nanopore sequencer (Serial No. MN24064) with a Flongle flow cell. Sequence data in FASTA format were submitted to NanoARG, an online computational resource that utilizes the long reads produced by nanopore sequencing technology. NanoARG offers various means to analyze and visualize data, including the simultaneous quantitative profiling of ARGs, mobile gene elements, and other information. ARG data were available from the output data, and the dataset was used to estimate AMR pollution.

## Results and discussion

### Resistome analysis of eDNA

**Figure 2:**
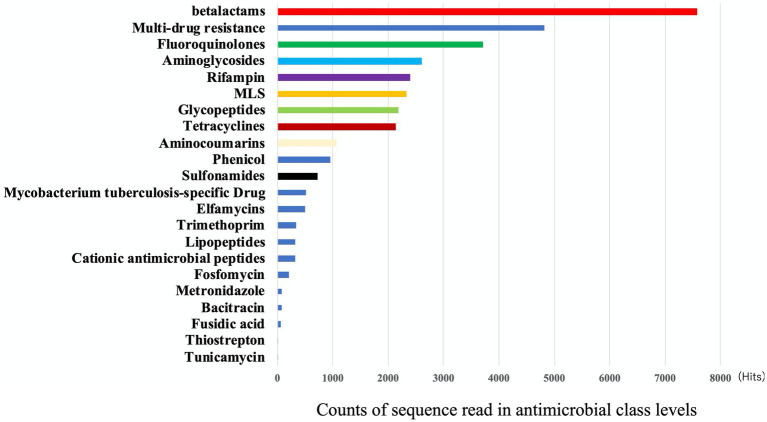
The frequency of AMR class detected in eDNA. Output results provided by AmrPlusPlus are shown as Class levels. The class levels are the major molecular category of resistance to different classes of antimicrobial drugs. Gene fraction is defined as the proportion of nucleotides in a reference sequence to which at least one read from the sequence data is aligned. Counts of aligned reads were recorded and this figure showed the results of class. Abbrebiation. MLS; macrolide-lincosamide-streptogramin B.

For the eDNA, the most frequent type of AMR by class was found to be beta-lactams (23%), followed by multidrug resistance (15%) and fluoroquinolones (11%) (Figure 2). The most frequent AMR class in Figure 2, beta-lactams, was subsequently divided into classes A, B, C, and D by mechanisms (Table 1). By mechanisms, multidrug efflux pumps, fluoroquinolone-resistant DNA topoisomerases, and class A beta-lactamases were the most frequent resistance in that order (Table 1). In this way, we gained further insight into the type of AMR present in environmental water in Japan.

**Table 1:** The frequency of the ARGs on the basis of the Mechanism in eDNA.

Class	Mechanism	Order of Frequency	Hits
Multidrug-resistance	Multidrug efflux pumps	1	3,806
Fluoroquinolones	Fluoroquinolone-resistant DNA topoisomerases	2	3,254
Beta-lactams	Class A beta-lactamases (penicillinase)	3	2,511
Rifampin	Rifampin-resistant beta-subunit of RNA polymerase RpoB	4	2049
Beta-lactams	Class B beta-lactamses (metallo-betalactamases)	5	1722
Beta-lactams	Class D beta-lactamase (Oxacillinase)	6	1,426
Beta-lactams	Class C beta-lactamase (AmpC type β-lactamases)	7	1,246
Tetracyclines	Tetracycline resistance major facilitator superfamily MFS efflux pumps	8	1,143
Aminocoumarin	Aminocoumarin-resistant DNA topoisomerases	9	1,003
Aminoglycoside	Aminoglycoside N-acetyltransferases	10	860

Top 10 of frequency of ARGs were output by AmrPlusPlus. Counts of aligned reads were recorded at the gene, group, mechanism, and class levels provided. This table was based on the Mechanism level. The Mechanism level is under the Class level and corresponds to molecular mechanism that confers resistance to antibiotics.

By ARGs group levels in beta-lactam, the OXA-group occurred most frequently, followed by the CTX-group (Table 2). Then, we summarized the most existing ARGs of the OXA-type carbapenemases in the environmental water in Table 3. We gained the results of 343 OXA-type genes, of which 30 were selected with sequence read counts of more than ten hits. These 30 genes were described by Assession no., source organism, and molecular type by referring to the National Center for Biotechnology Information (NCBI) database.

**Table 2:** The top 5 frequency of Beta-lactam group in eDNA.

Order	Group	Number of detected accessions in the group
1	OXA^*1^	343
2	CTX^*2^	184
3	SHV^*3^	127
4	CMY^*4^	104
5	TEM^*5^	101

This table indicated the detected number of ARGs Group levels in beta-lactams class. The number of detected accession in the group were summed up. Abbreviation: ^*1^OXA;oxacillinase, ^*2^CTX; cefotaximase, ^*3^SHV; Sulfhydryl variable, ^*4^CMY; AmpC variant and named CMY because of its phenotypic trait associated with cephamycinase and was resistant to cefoxitin. ^*5^TEM; one of beta-lactamase and named from the patient “Temoneira” from whom *E. coli*.

**Table 3:** The frequently detected OXA-type genes and the putative source organisms in eDNA.

Related gene	Source organism	Assession no	Mol_Type
OXA	*Burkholderia pseudomallei*	AJ488303.1	Genomic DNA
OXA	*Pseudomonas aeruginosa*	AF317511	Genomic DNA
OXA-15	*Pseudomonas aeruginosa*	U63835.1	Genomic DNA
OXA-18	*Pseudomonas aeruginosa*	EU503121.1	Genomic DNA
OXA-22	*Ralstonia pickettii*	AF064820	Genomic DNA
OXA-29	*Fluoribacter gormanii*	AJ400619.1	Genomic DNA
OXA-45	*Pseudomonas aeruginosa*	AJ519683, AM849110	Genomic DNA
OXA-50a	*Pseudomonas aeruginosa*	AY306130	Genomic DNA
OXA-53	*Salmonella enterica sub*sp. *enterica serovar Agona*	AY289608.1	Genomic DNA
OXA-55	*Shewanella algae*	AY343493	Genomic DNA
OXA-59	*Burkholderia pseudomallei*	AJ632249.1	Genomic DNA
OXA-60a	*Ralstonia pickettii*	AY662675	Genomic DNA
OXA-60c	*Ralstonia pickettii*	AY664505	Genomic DNA
OXA-60d	*Ralstonia pickettii*	AY664506	Genomic DNA
OXA-61	*Campylobacter jejuni*	AY587956	Genomic DNA
OXA-62	*Pandoraea pnomenusa*	AY423074	Genomic DNA
OXA-85	*Fusobacterium nucleatum*	AY227054.1	Genomic DNA
OXA-114e	*Achromobacter xylosoxidans*	HM104634	Genomic DNA
OXA-129	*Salmonella enterica subsp. enterica serovar Bredeney*	AM932669	Genomic DNA
OXA-134a	*Acinetobacter lwoffii*	HQ122933.1	Genomic DNA
OXA-198	*Pseudomonas aeruginosa*	HQ634775	Genomic DNA
OXA-205	*Pseudomonas aeruginosa*	JF800667.1	Genomic DNA
OXA-215	*Acinetobacter haemolyticus*	JN861783	Genomic DNA
OXA-243a	*Achromobacter xylosoxidans*	JX206446	Genomic DNA
OXA-258	*Achromobacter ruhlandii*	HE614014.2	Genomic DNA
OXA-309	*Acinetobacter johnsonii*	HF947514	Genomic DNA
OXA-333	*Acinetobacter johnsonii*	KF203107	Genomic DNA
OXA-334	*Acinetobacter johnsonii*	KF203108.1	Genomic DNA
OXA-335	*Acinetobacter lwoffii*	KF203109	Genomic DNA
OXA-362	*Acinetobacter lwoffii*	KF460532.1	Genomic DNA

Source organisms were searched by assession no. *via* the NCBI. We selected 30 OXA-type genes out of 343 genes, with sequence read counts of more than 10 hits. OXA; oxacillinase.

As far as OXY-type carbapenemases, it has been observed by Walther-Rasmussen and Høiby (2006) that there has been a significant increase in the number of class D beta-lactamase, which is believed to be an ancestor to one of the plasmid-encoded OXA-type carbapenemases. The OXA beta-lactamases were among the earliest ones discovered and always plasmid-mediated (Evans and Amyes, 2014). From the point of the One-Health approach, we considered it is crucial to understand the current pollution status of the OXA-type ARG of environmental water in Tokyo.

From our eDNA analysis, we have found frequent detection of OXA-type genes in Japanese environmental water. Table 3 indicates that some OXA-type carbapenemases are widely dispersed among *P. aeruginosa* and *Acinetobacter* strains. The source organisms listed include clinical pathogens such as *P. aeruginosa* and *Campylobacter jejuni*, as well as environmental bacteria such as *Ralstonia pickettii, Achromobacter xylosoxidans, Acinetobacter johnsonii*, and *Fluoribacter gormanii*. While clinical and environmental bacteria exist, we should pay attention to understanding this result. The annotation in the database only reflects results that have been studied and submitted to the database and is, therefore, not allocated for uncultured bacteria in the environmental water ecosystem. In carbapenem-hydrolyzing OXA-type carbapenemases, many variants are known. Especially, OXA-51 carbapenemase has many variants, known as OXA-51-like beta-lactamases (Evans and Amyes, 2014). However, we did not detect any OXA-51-like carbapenemase genes in eDNA samples. We have identified three different groups of enzymes: OXA-134a (OXA-134a-like group), OXA-215 (OXA-214-like group), and OXA-309 (OXA-211-like group). These groups are encoded in the chromosome (Evans and Amyes, 2014). In clinical settings, it is critically concerned with pathogenic ARGs, NDM-beta-lactamase (Toomer et al., 2020). It was widespread environmental contamination (Mills and Lee, 2019; Usman Qamar et al., 2020). Compared to the frequent ARGs listed in Table 1, it was low-frequency but very pathogenic ARGs, NDM-beta-lactamase were detected in our samples (Table 4). NDM-1 through NDM-10, NDM-12, NDM-13, and NDM-15 were confirmed by NCBI queries. NDM-5 is becoming a significant threat to Americans (Rojas et al., 2017). In summary, eDNA analysis indicated the frequent presence of pathogenic ARGs such as OXA-type AMR genes. Also, we could find clinically threatening NDM beta-lactamase in the river of Tokyo, although it was not frequent.

**Table 4:** Detected New Delhi Metallo (NDM) beta-lactamase genes in eDNA.

Group	Gene names	Assession no.	Organisms	Gene Fraction	Hits
NDM	blaNDM-1	KC149527	*Escherichia coli plasmid*	6.27306	4
NDM	blaNDM-5	JN104597	*Escherichia coli*	5.78106	4
NDM	blaNDM-9	KC999080	*Klebsiella pneumoniae plasmid*	2.70603	2
NDM	blaNDM-13	LC012596	*Escherichia coli plasmid*	6.15006	4
NDM	blaNDM-12	AB926431	*Escherichia coli plasmid*	11.5621	7
NDM	blaNDM-2	JN112341	*Acinetobacter baumannii*	9.47109	6
NDM	blaNDM-6	JN967644	*Escherichia coli plasmid*	4.18204	3
NDM	blaNDM-7	JX412225	*Escherichia coli*	7.74908	5
NDM	blaNDM-1	FN396876	*Klebsiella pneumoniae plasmid*	6.14251	4
NDM	blaNDM-1	JF826285	*Klebsiella pneumoniae*	1.79558	1
NDM	blaNDM-4	JQ348841	*Escherichia coli*	4.67405	3
NDM	blaNDM-3	JQ734687	*Escherichia coli*	7.50308	5
NDM	blaNDM-8	AB744718	*Escherichia coli plasmid*	11.3161	7
NDM	blaNDM-10	KF361506	*Klebsiella pneumoniae*	6.51907	5
NDM	blaNDM-15	KP735848	*Escherichia coli*	5.90406	4

NDM genes were detected in eDNA. Gene names and organisms were confirmed by NCBI query with Assession no.

### Antimicrobial susceptibility of isolates

We subjected a total of 284 isolates to antimicrobial susceptibility testing with six antimicrobials (i.e., amikacin, tetracycline, ampicillin-sulbactam, levofloxacin, imipenem, and clarithromycin). The categories of “resistant” and “intermediate” were interpreted as AMR, and we calculated the AMR ratio (%). A high AMR ratio was detected for clarithromycin (CLR15; 75%), tetracycline (TE10; 53%), and ampicillin-sulbactam (SAM20; 48%) (Table 5). Then, we selected 22 multidrug-resistant isolates with more than two “resistant” antimicrobials as listed in Table 6. We referred to NanoARG algorithms or EPI2ME WIMP software (Nanopore website) for bacteria species. The sequences were cross-checked on the NCBI website using the BLAST function. Environmental bacteria and pathogenic bacteria were listed as multidrug-resistant bacteria.

**Table 5:** Summary of antimicrobial susceptibility testing was performed using the BBL™ Sensi-Disc™ susceptibility testing kit (Becton Dickinson) on Mueller–Hinton agar (Becton Dickinson.

Antimicrobials	TE10	SAM20	AN30	LVX5	IPM10	CLR15
Resistant (R)	80	112	57	21	34	170
Intermediate (I)	71	25	18	19	23	39
Total sum (R + I)	151	137	75	40	57	209
Tested isolates	284	284	284	284	284	284
AMR ration (%)	53%	48%	26%	14%	20%	75%

The antimicrobial agents were used six antimicrobials; amikacin 30 μg (AN30), tetracycline 30 μg (TE30), ampicillin-sulbactam 10/10 μg (SAM20), levofloxacin 5 μg (LVX5), imipenem 10 μg (IPM10), and clarithromycin 15 μg (CLR15). Then inhibition zone diameters were measured to classify the degree of susceptibility. All isolates were classified as sensitive (S), intermediate (I), and resistant (R) according to the manufacturer’s provided standard for *Enterobacteriaceae, P. aeruginosa, Acinetobacter*, and *Staphylococci*. This standard is based on the Clinical and Laboratory Standards Institute [CLSI] (2018) breakpoints. All the data are available in Supplementary Table S1.

**Table 6:** Results of antimicrobial susceptibility testing of high-resistant isolates.

Sampling St.	Sample ID	TE30	SAM20	AN30	LVX5	IPM10	CLR15	Identified bacteria species	Additional info
St1	92	R	R	R	S	S	R	*Raoultella ornithinolytica*	Environment, histamine poisoning
St33	102	R	R	S	S	S	R	*Escherichia coli*	Lower gut of animals and survives even in the natural environment.
St33	103	I	R	S	S	R	R	*Kosakonia cowanii*	Usually recognized as a plant pathogen
St35	114	I	R	R	R	R	R	*Escherichia coli*	Lower gut of animals and survives even in the natural environment.
St38	120	R	R	S	R	S	R	*Serratia marcescens*	Environment, rarely reported as an opportunistic pathogen
St4	125	R	R	R	S	R	R	*Escherichia coli*	Lower gut of animals and survives even in the natural environment.
St7	204	I	R	S	S	R	R	*Pseudomonas putida*	Environment, rarely reported as an opportunistic pathogen
St57	211	I	R	S	S	S	R	*Serratia liquefaciens*	Environment, rarely reported as an opportunistic pathogen
St45	227	S	R	S	S	S	R	*Raoultella ornithinolytica*	Found in water environments and soil, histamine poisoning in humans.
St45	230	S	R	S	S	S	R	*Pseudomonas mosselii*	A novel species, which has been characterized in 2002 (Dabboussi F et al, 2002)regarded as a potential pathogen.
St37	234	R	S	R	R	S	R	*Pectobacterium carotovorum*	Plant pathogen Gram-negative bacterium (soft rot disease of potato tubers).
St38	245	S	I	S	S	R	R	*Klebsiella pneumoniae*	The species K. pneumonia, are opportunistic pathogens that can cause pneumonia
St38	247	S	R	S	S	S	R	*Pseudomonas protegens*	Pseudomonas protegens sp. nov., widespread plant-protecting bacteria producing the biocontrol compounds
St55	256	I	R	R	I	S	R	*Bacillus cereus*	It is responsible for food poisoning outbreaks.
St54	265	I	S	S	S	R	R	*Enterobacter cloacae*	*Enterobacter cloacae* is a prevalent nosocomial pathogen as it is highly resistant to disinfectants and antimicrobial agents.
St42	294	I	R	S	I	S	R	*Rahnella* sp. *Y9602*	A facultatively anaerobic, nitrogen-fixing, Gram-negative bacterium. Found in fresh water and human intestinal microflora.
St43	304	S	I	R	S	S	R	*Bacillus pumilus*	Environment.
St23	312	S	R	S	S	R	R	*Rahnella* sp. *Y9602*	A facultatively anaerobic, nitrogen-fixing, Gram-negative bacterium. Found in fresh water and human intestinal microflora.
St22	314	S	S	R	R	S	R	*Serratia fonticola*	An unusual human pathogen. Previously described primarily as causing skin and soft tissue infections following trauma.
St22	332	R	R	R	R	R	R	*Pantoea vagans*	A gram-negative enterobacterial plant epiphyte isolated from apple, and an important biocontrol agent as Blight Ban C9-1.
St24	340	S	R	S	S	S	R	*Raoultella ornithinolytica*	Found in water environments and soil, histamine poisoning in humans.
St26	346	S	I	R	S	R	R	*Proteus vulgaris*	*Proteus vulgaris* account for most clinical Proteus isolates.

Twenty-two isolates were selected as multidrug-resistant bacteria. Bacterial species were identified using NanoARG algorithms or EPI2ME WIMP software (Nanopore website). The sequences were cross-checked on the NCBI website using the BLAST function. Abbreviation: resistant (R), Intermediate (I), Sensitive (S); These criteria were determined according to the instruction of the BBL™ Sensi-Disc™ susceptibility testing kit.

### Amr profile of the ARG class of multidrug-resistant bacteria (mrDNA)

**Figure 3:**
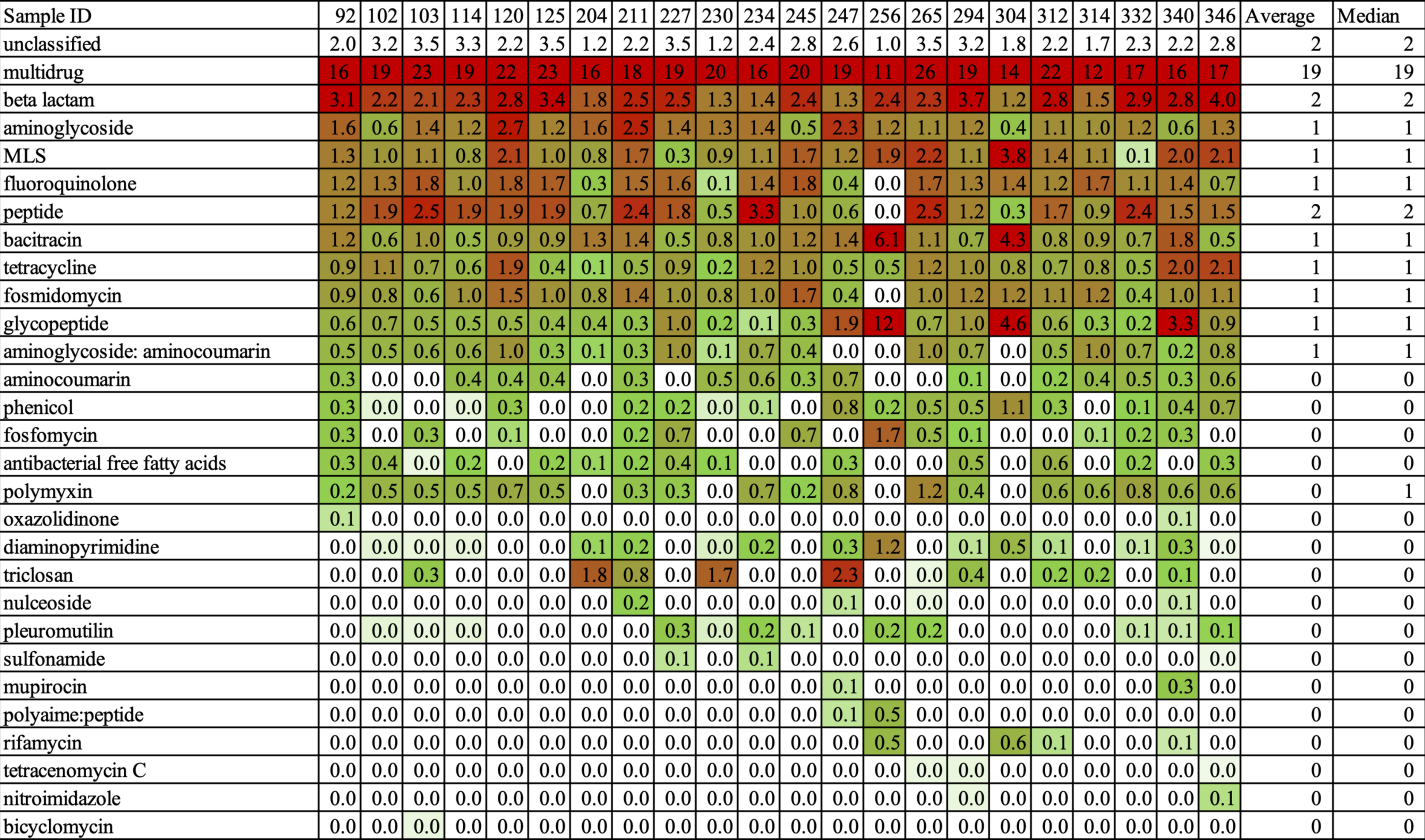
The Heatmap about the abundance of AMR classes in high-resistant bacteria. This Heatmap was output by NanoARG. Abundance of ARG classes is estimated by the copy number of ARGs. The copy number of ARGs is normalized to the total gigabase pairs (Gbp) of the sample. There, the relative ARG abundances could compare to the content of ARGs among the samples. NanoARG used the DeepARG-LS model, which is applied with permissive parameters, an identity cutoff of 25%, a coverage of 40%, and a probability of 0.5, to predict. Red indicated high abundance and green showed a low abundance.

The abundance of ARG classes was estimated by the copy number of ARGs based on NanoARG algorithms. The copy numbers of ARGs were normalized to the total gigabase pairs of the sample to obtain the relative ARG abundance (Ma et al., 2016). After normalization, we could compare the abundance of ARGs across samples. The result is shown in Figure 3. In this heat map, red indicates high abundance and green indicates low abundance. As for the mrDNA data, the most frequent ARG class was multidrug resistance, followed by beta-lactam resistance (Figure 3). The abundance of the multidrug class was 19/Gbp on average (median = 19) and thus far higher than the abundance of the beta-lactam class at 2/Gbp on average (median = 2), resulting in a 9.5-fold increase. Figure S1 shows the sequence reads of the ARG classes for each sample, and again, the multidrug class far exceeded all other classes in abundance. This result implied that earning multidrug-resistant functions such as multidrug efflux pumps is essential to survive in environmental water. It is a fascinating insight from the view of understanding of the bacterial ecosystem. However, it is not the best way to catch various AMR genes in the aim of this study.

Establishing a clear relationship between antimicrobial susceptibility testing and AMR genes in mrDNA analysis was challenging. Sample ID 340 was resistant to SAM20 and CLR15, while Sample ID 332 was resistant to all antimicrobial agents, as indicated in Table 6. This suggests that Sample ID 332 was more multidrug-resistant compared to Sample ID 340. In terms of AMR genes, Sample ID 340 had more AMR genes compared to Sample ID 332, as shown in Figure 3. The characterization of antimicrobial resistance did not coincide with the types of AMR genes found in mrDNA in environmental water. This is a reasonable result, considering their AMR profiles. The most frequently found AMR gene is multidrug-resistant, which can work against many types of drugs. Therefore, we thought it was not a suitable sample for understanding comprehensive AMR pollution in environmental water using mrDNA analysis.

### Effective AMR pollution analysis of environmental water samples

Our study consisted of two experiments that analyzed eDNA and mrDNA. eDNA was obtained from water samples using a Sterivex filter and includes all types of DNA - culturable, unculturable, and exo-cellular. Since the Sterivex filter can capture DNA from both dead and alive cells, it provides an overview of AMR pollution in water. On the other hand, mrDNA was extracted from isolated bacteria using a culture-dependent method during the screening process. It is known that 99% of environmental bacteria are challenging to culture on artificial media, making mrDNA effective only on culturable-isolated bacteria. This is the primary difference between eDNA and mrDNA. The most significant difference in results between the two was the frequently detected ARGs. Beta-lactams were the most common in eDNA samples, while multidrug ARGs were present in mrDNA. For effective targeting of antimicrobial-resistant contamination, it is essential to have specific information about antimicrobial-resistant ARGs, such as beta-lactams, rather than non-specific multidrug ARGs. On this point, eDNA is proposed as a more reliable source of information than mrDNA. This result implies that eDNA is a better indicator than mrDNA in the presence of AMR in the water environment.

In the process of eDNA, a Sterivex filter was often used (Miya et al., 2020). There are two merits. Firstly, it reduces the laboratory’s contamination probability because the Sterivex-filter is individually packaged and sterile. Secondly, it could capture uncultured bacterial DNA. Meanwhile, when using eDNA for monitoring AMR pollution, a large amount of sample water is needed because the amount of eDNA in the river is low concentration. In order to establish a simple and affordable way of monitoring, it is necessary to devise effective techniques for concentrating DNA in water. Most environmental bacteria, that is, uncultured bacteria, can contribute to preserving drug-resistance genes in the water environment. Therefore, the sample must include uncultured environmental bacteria such as eDNA.

Another new technique called environmental RNA (eRNA) is used to study the ecology of environmental water, as explained by Yates and colleagues (Yates et al., 2021). One of its significant advantages is its ability to detect unique eRNA signatures of organisms beyond the species level, thus improving identification accuracy. However, RNA is less stable than DNA. Despite being a relatively new study area, environmental RNA is expected to have great potential for biomonitoring. Further research is needed to explore its potential for monitoring AMR by RNA in environmental water at the present.

As for the metagenomics tool, both Illumina and MinIon worked well. However, the Illumina platform is time-consuming in the procedure’s pretreatment and is more expensive for the reagent kit and device than the MinIon platform. Data handling in NanoARG, designed for the MinIon platform, also works more user-friendly than AmrPlusPlus. From acquiring sequence data to the output of AMR genes, it is possible to deal with big-sequenced data smoothly. Therefore, using MinION and NanoARG together would be preferable based on our current findings.

This study focuses on the monitoring method, especially the appropriate sample detecting AMR pollution in environmental river using NGS and bioinformatic techniques. Our data suggested that eDNA is more appropriate sample than mrDNA to catch various ARGs in environmental water. However, there remains some improvement in eDNA. The standard monitoring for environmental ARGs should be an easy-handling method. In the point, eDNA is not concentrated in nature. To this end, developing the concentrated eDNA technique from environmental water would be expected.

## Conclusion

We conducted a study using both eDNA and mrDNA methods to examine the current state of AMR contamination in environmental water samples in the Tokyo area. Our findings reveal that β-lactams and multidrug-resistant ARGs were frequently detected. The eDNA analysis identified ARGs for beta-lactams, fluoroquinolones, and aminoglycosides more frequently than the mrDNA analysis. We also detected various types of NDM-type genes, which are critical pathogens and require regular monitoring. The mrDNA analysis detected more multidrug ARGs than specific-drug ARGs. Furthermore, non-specific ARGs that confer multidrug resistance in pathogenic bacteria were detected at a higher rate (9.5-fold) than other antibacterial agents such as β-lactams. These results suggested that multidrug resistance mechanisms in environmental bacteria differ from those in clinical settings. Our study highlights the importance of eDNA analysis in understanding the spread of antimicrobial resistance in environmental waters.

## Data availability statement

The datasets presented in this study can be found in online repositories. The names of the repository/repositories and accession number(s) which the sequence data is available in is NCBI with Biosample Accession numbers: SSUB027355 (SAMD00653937 - SAMD00653958), SSUB027355, eDNA(SAMD00653936).

## Author contributions

KT-N: most experiment and manuscript writing. RP: mrDNA experiment, data analysis, and manuscript editing. NM: Field work and sampling water treatment. TS: total study design and data analysis. All authors contributed to the article and approved the submitted version.

## References

[ref1] Arango-Argoty G. A. Dai D. Pruden A. Vikesland P. Heath L. S. Zhang L. (2019). NanoARG: a web service for detecting and contextualizing antimicrobial resistance genes from nanopore-derived metagenomes. Microbiome 7:88. doi: 10.1186/s40168-019-0703-9, PMID: 31174603 PMC6555988

[ref2] Arango-Argoty G. Garner E. Pruden A. Heath L. S. Vikesland P. Zhang L. (2018). DeepARG: a deep learning approach for predicting antibiotic resistance genes from metagenomic data. Microbiome 6:23. doi: 10.1186/s40168-018-0401-z, PMID: 29391044 PMC5796597

[ref1001] Dabboussi F. Hamze M. Singer E. Geoffroy V. Jean-Marie M. Daniel I. (2002). Pseudomonas mosselii sp. nov., a novel species isolated from clinical specimensa. Int J Syst Evol Microbiol. 52, 363–376. doi: 10.1099/00207713-52-2-363, PMID: 11931144

[ref3] Doster E. Lakin S. M. Dean C. J. Wolfe C. Young J. G. Boucher C.. (2020). MEGARes 2.0: a database for classification of antimicrobial drug, biocide and metal resistance determinants in metagenomic sequence data. Nucleic Acids Res. 48, D561–D569. doi: 10.1093/nar/gkz1010, PMID: 31722416 PMC7145535

[ref4] Evans B. A. Amyes S. G. B. (2014). OXA β-Lactamases. Clin. Microbiol. Rev. 27, 241–263. doi: 10.1128/CMR.00117-13, PMID: 24696435 PMC3993105

[ref5] Hassoun-Kheir N. Stabholz Y. Kreft J. U. de la Cruz R. Romalde J. L. Nesme J.. (2020). Comparison of antibiotic-resistant bacteria and antibiotic resistance genes abundance in hospital and community wastewater: a systematic review. Sci. Total Environ. 743:140804. doi: 10.1016/j.scitotenv.2020.140804, PMID: 32758846

[ref6] Iwane T. Urase T. Yamamoto K. (2001). Possible impact of treated wastewater discharge on incidence of antibiotic resistant bacteria in river water. Water Sci. Technol. 43, 91–99. doi: 10.2166/wst.2001.0077, PMID: 11380211

[ref7] Lakin S. M. Dean C. Noyes N. R. Dettenwanger A. Ross A. S. Doster E.. (2017). MEGARes: an antimicrobial resistance database for high throughput sequencing. Nucleic Acids Res. 45, D574–D580. doi: 10.1093/nar/gkw1009, PMID: 27899569 PMC5210519

[ref8] Liguori K. Keenum I. Davis B. C. Calarco J. Milligan E. Harwood V. J.. (2022). Antimicrobial resistance monitoring of water environments: a framework for standardized methods and quality control. Environ. Sci. Technol. 56, 9149–9160. doi: 10.1021/acs.est.1c08918, PMID: 35732277 PMC9261269

[ref9] Ma L. Xia Y. Li B. Yang Y. Li L.-G. Tiedje J. M.. (2016). Metagenomic assembly reveals hosts of antibiotic resistance genes and the shared Resistome in pig, chicken, and human feces. Environ. Sci. Technol. 50, 420–427. doi: 10.1021/acs.est.5b03522, PMID: 26650334

[ref10] Mackenzie J. S. Jeggo M. (2019). The one health approach-why is it so important? Trop Med Infect Dis 4:88. doi: 10.3390/tropicalmed4020088, PMID: 31159338 PMC6630404

[ref11] Mikheyev A. S. Tin M. M. Y. (2014). A first look at the Oxford Nanopore MinION sequencer. Mol. Ecol. Resour. 14, 1097–1102. doi: 10.1111/1755-0998.12324, PMID: 25187008

[ref12] Mills M. C. Lee J. (2019). The threat of carbapenem-resistant bacteria in the environment: evidence of widespread contamination of reservoirs at a global scale. Environ. Pollut. 255:113143. doi: 10.1016/j.envpol.2019.113143, PMID: 31541827

[ref13] Miya M. Gotoh R. O. Sado T. (2020). MiFish metabarcoding: a high-throughput approach for simultaneous detection of multiple fish species from environmental DNA and other samples. Fish. Sci. 86, 939–970. doi: 10.1007/s12562-020-01461-x

[ref14] Nakayama T. Tuyet Hoa T. T. Harada K. Warisaya M. Asayama M. Hinenoya A.. (2017). Water metagenomic analysis reveals low bacterial diversity and the presence of antimicrobial residues and resistance genes in a river containing wastewater from backyard aquacultures in the Mekong Delta, Vietnam. Environ. Pollut. 222, 294–306. doi: 10.1016/j.envpol.2016.12.041, PMID: 28062224

[ref15] OECD (2018). Stemming the superbug tide: just a few dollars more, OECD health policy studies. Paris: OECD Publishing. Available at: https://www.oecd-ilibrary.org/social-issues-migration-health/stemming-the-superbug-tide_9789264307599-en

[ref16] Qin J. Li R. Raes J. Arumugam M. Burgdorf K. S. Manichanh C.. (2010). A human gut microbial gene catalogue established by metagenomic sequencing. Nature 464, 59–65. doi: 10.1038/nature08821 20203603 PMC3779803

[ref17] Rojas L. J. Hujer A. M. Rudin S. D. Wright M. S. Domitrovic T. N. Marshall S. H.. (2017). NDM-5 and OXA-181 Beta-lactamases, a significant threat continues to spread in the Americas. Antimicrob. Agents Chemother. 61:e00454-17. doi: 10.1128/AAC 28461314 PMC5487671

[ref18] Ruppert K. M. Kline R. J. Rahman M. S. (2019). Past, present, and future perspectives of environmental DNA (eDNA) metabarcoding: a systematic review in methods, monitoring, and applications of global eDNA. Glob. Ecol. Conserv. 17:e00547. doi: 10.1016/j.gecco.2019.e00547

[ref19] Shin H. Kim Y. Han S. Hur H.-G. (2023). Resistome study in aquatic environments. J. Microbiol. Biotechnol. 33, 277–287. doi: 10.4014/jmb.2210.10044, PMID: 36655280 PMC10084755

[ref20] Taing L. Bhatia H. Kaiser R. A. Qadir M. Mehmood H. (2022). A rapid review of environmental health gaps in antimicrobial resistance and water-related research from 1990-2020. Int. J. Environ. Res. Public Health 19:6549. doi: 10.3390/ijerph19116549, PMID: 35682132 PMC9180282

[ref21] Takahara T. Minamoto T. Yamanaka H. Doi H. Kawabata Z. (2012). Estimation of fish biomass using environmental DNA. PLoS One 7:e35868. doi: 10.1371/journal.pone.0035868, PMID: 22563411 PMC3338542

[ref22] Toomer K. H. de Lima Corvino D. McCrink K. A. Gonzales Zamora J. A. (2020). A New Delhi metallo-β-lactamase (NDM)-positive isolate of *Klebsiella pneumoniae* causing catheter-related bloodstream infection in an ambulatory hemodialysis patient. IDCases 21:e00816. doi: 10.1016/j.idcr.2020.e00816, PMID: 32461908 PMC7242868

[ref23] Usman Qamar M. S Lopes B. Hassan B. Khurshid M. Shafique M. Atif Nisar M.. (2020). The present danger of New Delhi metallo-β-lactamase: a threat to public health. Future Microbiol. 15, 1759–1778. doi: 10.2217/fmb-2020-0069, PMID: 33404261

[ref24] Walther-Rasmussen J. Høiby N. (2006). OXA-type carbapenemases. J. Antimicrob. Chemother. 57, 373–383. doi: 10.1093/jac/dki482 16446375

[ref25] Weinstein M. P. (2018). M100-performance standards for antimicrobial susceptibility testing. 28th Edn Clinical and Laboratory Standards Institute.

[ref26] Wong M. K. S. Nakao M. Hyodo S. (2020). Field application of an improved protocol for environmental DNA extraction, purification, and measurement using Sterivex filter. Sci. Rep. 10:21531. doi: 10.1038/s41598-020-77304-7, PMID: 33298993 PMC7725969

[ref27] Yates M. C. Derry A. M. Cristescu M. E. (2021). Environmental RNA: A Revolution in Ecological Resolution? Trends Ecol. Evol. 36, 601–609. doi: 10.1016/j.tree.2021.03.001, PMID: 33757695

[ref28] Young H. -K. Jesudason M. V. (1990). Antibiotic resistant BACTERIA in drinking water in South India. J. Pharm. Pharmacol. 42:66P. doi: 10.1111/j.2042-7158.1990.tb14439.x

[ref29] Young S. Juhl A. O’Mullan G. D. (2013). Antibiotic-resistant bacteria in the Hudson River estuary linked to wet weather sewage contamination. J. Water Health 11, 297–310. doi: 10.2166/wh.2013.131, PMID: 23708577

[ref30] Zhou Z.-C. Zheng J. Wei Y.-Y. Chen T. Dahlgren R. A. Shang X.. (2017). Antibiotic resistance genes in an urban river as impacted by bacterial community and physicochemical parameters. Environ. Sci. Pollut. Res. 24, 23753–23762. doi: 10.1007/s11356-017-0032-0, PMID: 28864929

