# Supplementary material for: What samples are suitable for monitoring antimicrobial-resistant genes? Using NGS technology, a comparison between eDNA and mrDNA analysis from environmental water
Source: Front Microbiol. 2023 Dec 14;14:954783. doi: 10.3389/fmicb.2023.954783 (PMC10765985; doi:10.3389/fmicb.2023.954783)
Supplement: Supplementary file 1 [file Image_1.pdf]

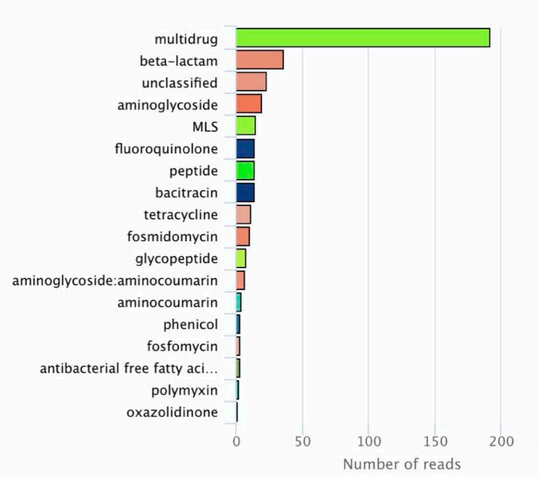

Sample ID92

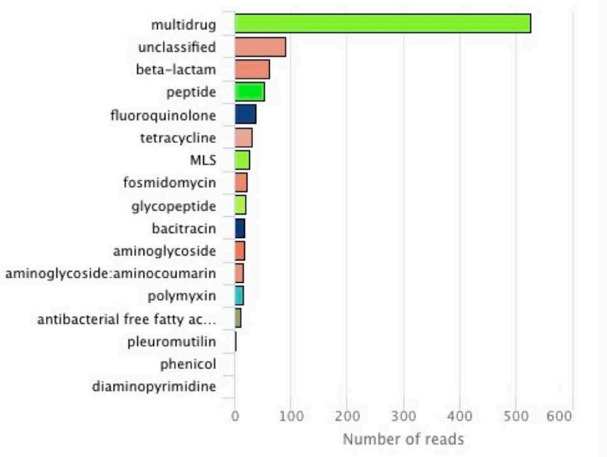

Sample ID102

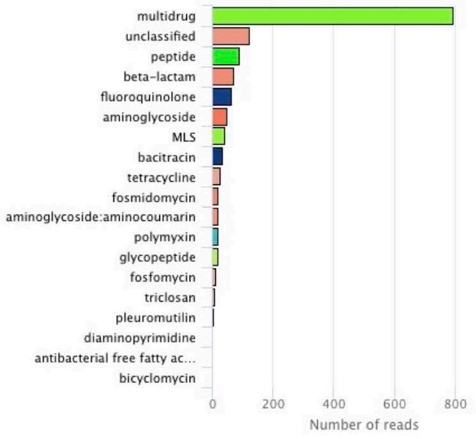

Sample ID103

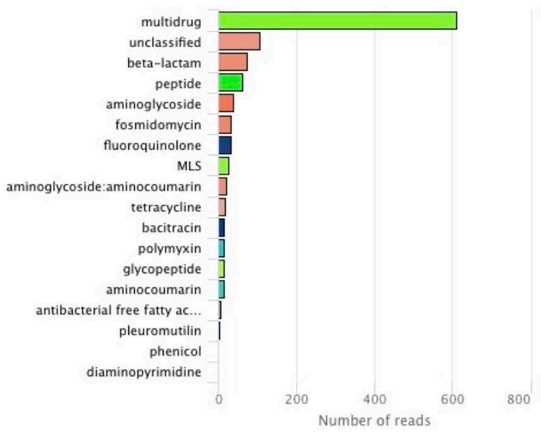

Sample ID114

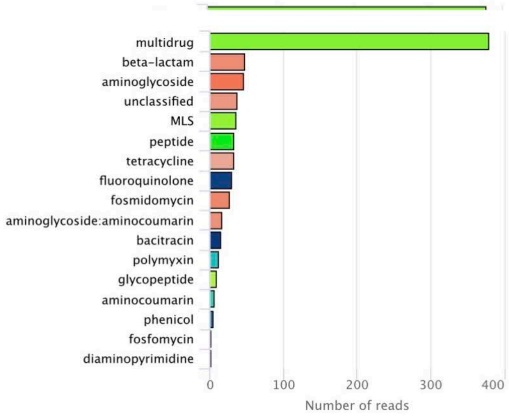

Sample ID120

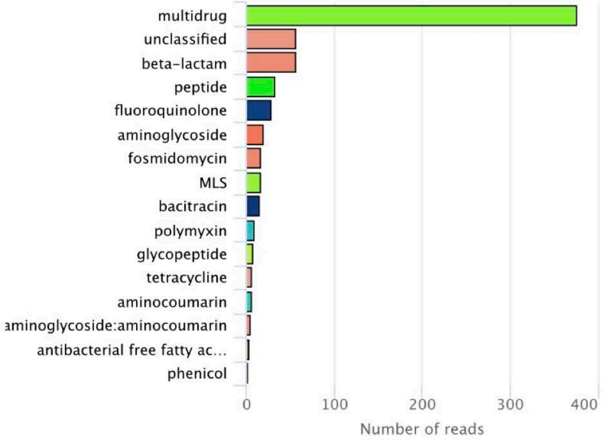

Sample ID125

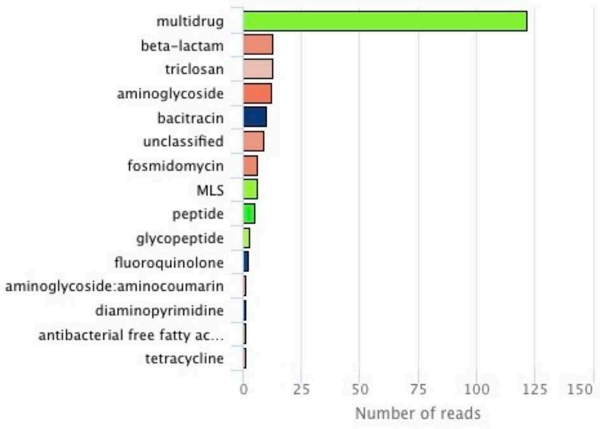

Sample ID204

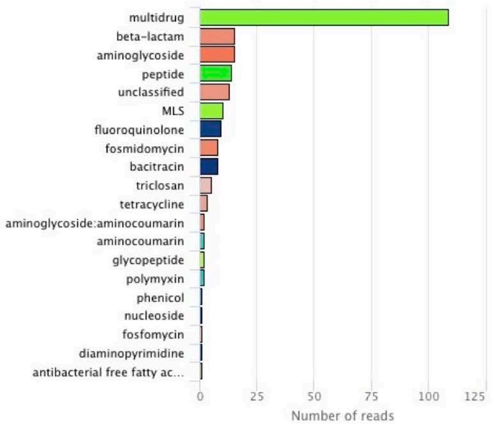

Sample ID211

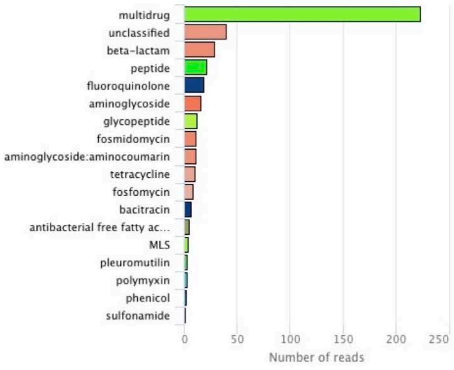

Sample ID227

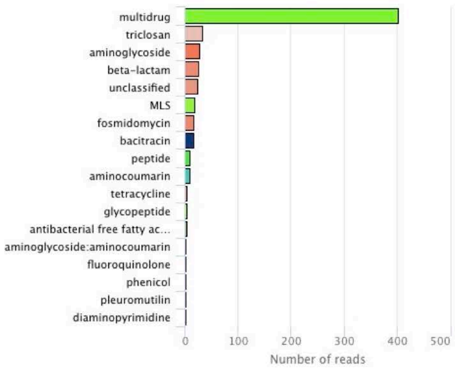

Sample ID230

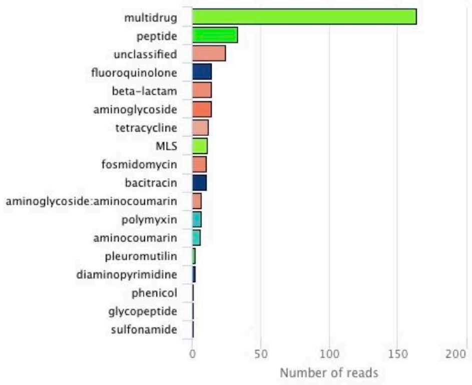

Sample ID234

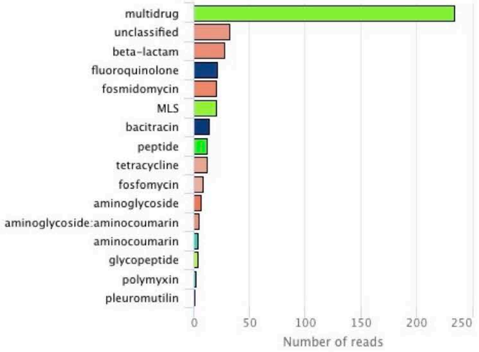

Sample ID245

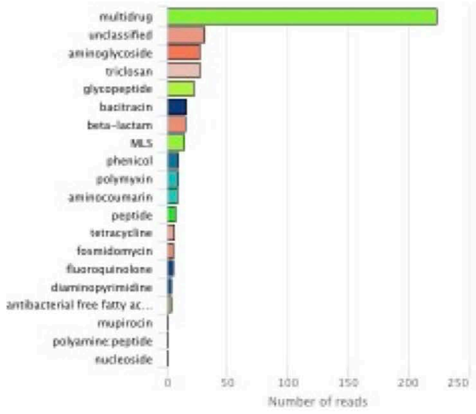

Sample ID247

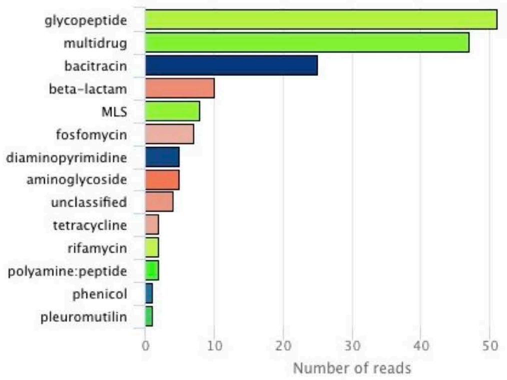

Sample ID256

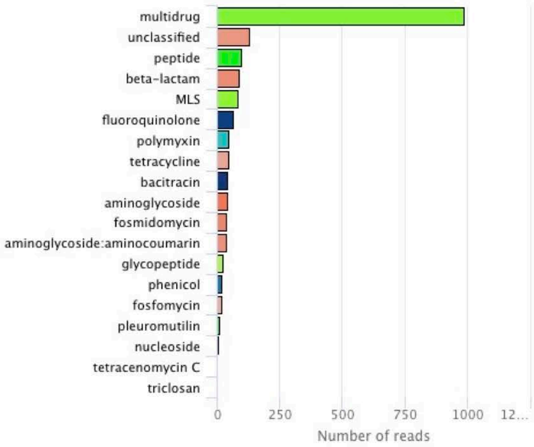

Sample ID265

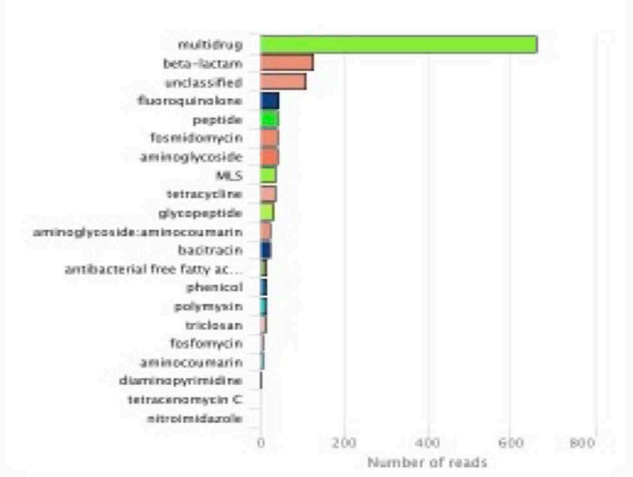

Sample ID294

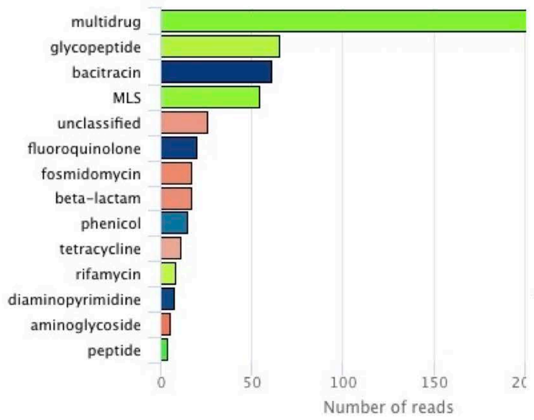

Sample ID304

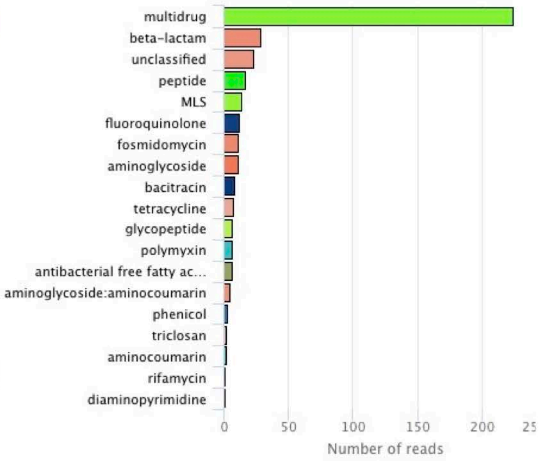

Sample ID312

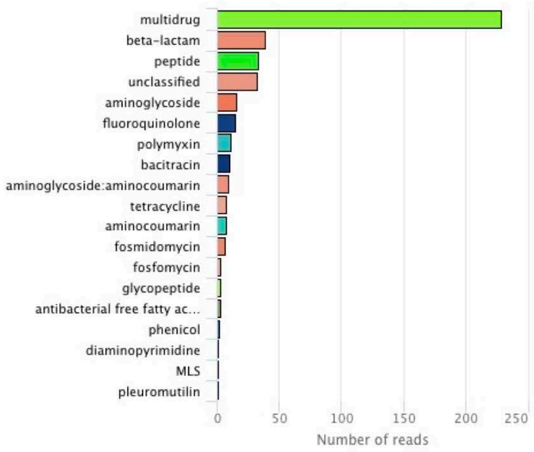

Sample ID332

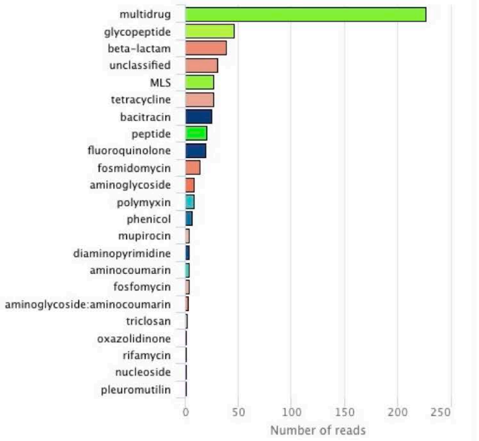

Sample ID340

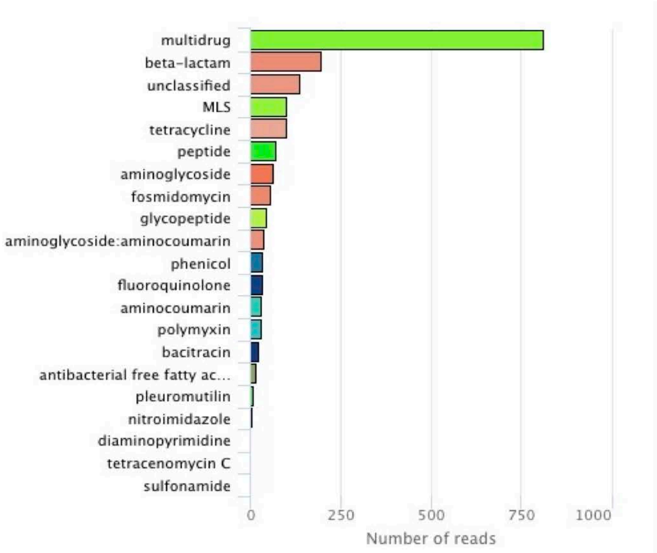

Sample ID346
